# Early specific cognitive-behavioural psychotherapy in subjects at high risk for bipolar disorders: study protocol for a randomised controlled trial

**DOI:** 10.1186/1745-6215-15-161

**Published:** 2014-05-08

**Authors:** Andrea Pfennig, Karolina Leopold, Andreas Bechdolf, Christoph U Correll, Martin Holtmann, Martin Lambert, Carolin Marx, Thomas D Meyer, Steffi Pfeiffer, Andreas Reif, Maren Rottmann-Wolf, Natalie M Schmitt, Thomas Stamm, Georg Juckel, Michael Bauer

**Affiliations:** 1 Department of Psychiatry and Psychotherapy, Carl Gustav Carus University Hospital, Technische Universität Dresden, Fetscherstrasse 74, 01307 Dresden, Germany; 2 Department of Psychiatry, Psychotherapy and Psychosomatics, Vivantes Hospital Urban, Berlin, Germany; 3 Department of Psychiatry and Psychotherapy, University Hospital Cologne, Cologne, Germany; 4 Division of Psychiatry Research, The Zucker Hillside Hospital, Glen Oaks, NY, USA; 5 Child and Adolescent Psychiatry, LWL-University Hospital for Child and Adolescent Psychiatry, Ruhr-University Bochum, Hamm, Germany; 6 Psychosis Centre, Department of Psychiatry and Psychotherapy, Center of Psychosocial Medicine, University Medical Centre Hamburg-Eppendorf, Hamburg, Germany; 7 Institute of Neuroscience, Newcastle University, Newcastle upon Tyne, UK; 8 Department of Psychiatry, Psychosomatics and Psychotherapy, University Hospital Würzburg, Würzburg, Germany; 9 Department of Psychiatry and Psychotherapy, Charite-University Medicine Berlin, Berlin, Germany; 10 LWL University Hospital and Department of Psychiatry, Psychotherapy and Preventive Medicine, Ruhr University Bochum, Hamm, Germany

**Keywords:** Bipolar disorders, Early recognition, Early intervention, Cognitive-behavioural psychotherapy, Intervention study, Randomised controlled trial

## Abstract

**Background:**

Bipolar disorders (BD) are among the most severe mental disorders with first clinical signs and symptoms frequently appearing in adolescence and early adulthood. The long latency in clinical diagnosis (and subsequent adequate treatment) adversely affects the course of disease, effectiveness of interventions and health-related quality of life, and increases the economic burden of BD. Despite uncertainties about risk constellations and symptomatology in the early stages of potentially developing BD, many adolescents and young adults seek help, and most of them suffer substantially from symptoms already leading to impairments in psychosocial functioning in school, training, at work and in their social relationships. We aimed to identify subjects at risk of developing BD and investigate the efficacy and safety of early specific cognitive-behavioural psychotherapy (CBT) in this subpopulation.

**Methods/Design:**

EarlyCBT is a randomised controlled multi-centre clinical trial to evaluate the efficacy and safety of early specific CBT, including stress management and problem solving strategies, with elements of mindfulness-based therapy (MBT) versus unstructured group meetings for 14 weeks each and follow-up until week 78. Participants are recruited at seven university hospitals throughout Germany, which provide in- and outpatient care (including early recognition centres) for psychiatric patients. Subjects at high risk must be 15 to 30 years old and meet the combination of specified affective symptomatology, reduction of psychosocial functioning, and family history for (schizo)affective disorders. Primary efficacy endpoints are differences in psychosocial functioning and defined affective symptomatology at 14 weeks between groups. Secondary endpoints include the above mentioned endpoints at 7, 24, 52 and 78 weeks and the change within groups compared to baseline; perception of, reaction to and coping with stress; and conversion to full BD.

**Discussion:**

To our knowledge, this is the first study to evaluate early specific CBT in subjects at high risk for BD. Structured diagnostic interviews are used to map the risk status and development of disease. With our study, the level of evidence for the treatment of those young patients will be significantly raised.

**Trial registration:**

WHO International Clinical Trials Platform (ICTRP), identifier: DRKS00000444, date of registration: 16 June 2010.

## Background

Bipolar disorders (BD) are among the most severe mental disorders. Lifetime prevalence estimates range from 1 to 5% [1-5]. They are associated with a recurrent or chronic course, insufficient clinical response, and psychosocial impairment in a substantial number of patients. First clinical signs and symptoms frequently appear in adolescence and early adulthood (median age at disease onset 17.5 years) [6-8]. Usually there is, however, a long lag before the correct diagnosis is established and treatment is often delayed for many years [6,7,9-13]. Delayed treatment and an increasing number of illness episodes have been associated with a decreased probability of response to treatment [14] and an adverse course of illness [15,16]. The combination of long undetected illness with no or inadequate treatment and significant psychosocial impairment renders early identification and intervention a vital role in disease management.

To date, there has been relatively little research into early identification and intervention in subjects at risk for BD. First at-risk criteria based on clinical presentation and/or family history have been proposed and pilot evaluation data are available [17,18]. Moreover, structured diagnostic instruments for the prospective identification of at-risk constellations for BD have been developed and are currently validated [19,20]. These structured measures are applied in the present study. Regarding treatment in these at-risk states, in a recent systematic review [21] we identified three studies: an exploratory, controlled study of multi-family psycho-educational psychotherapy from the group of Fristad and colleagues [22], an open, uncontrolled easibility study of family-focussed therapy by Miklowitz *et al*. [23] and the subsequent randomised controlled study of the same group [24]. Treatment with the studied interventions (in addition to treatment as usual, including psychopharmacology) showed a potential for symptom reduction and prevention of conversion to BD and a significantly faster recovery from initial mood symptoms and more time in remission during follow-up compared to control conditions. The results, however, have to be interpreted with caution, because of the small sample sizes, permission of medication treatments in the intervention and control groups in all studies, and the lack of a control group or data on conversion in one study. Until now, there is no data on the efficacy of early cognitive-behavioural psychotherapy (CBT) in high-risk subjects for BD. In current clinical routine, neither screening for at-risk states nor specialised diagnostic processes are implemented, and even in cases that present with significant affective symptoms, no specific intervention is routinely offered before the full disorder manifests.

### Objectives/hypotheses

CBT has been shown to be effective in BD (see [25,26]). Previous research suggested that, although there was no significant difference in overall recurrence rates, CBT is more effective than treatment as usual in bipolar patients with few, as compared to those with many, episodes in their history [27]. We therefore hypothesise that the intervention might be more effective in the early stages of disease and even more so in the prodromal phase.

We therefore aimed to conduct a prospective randomised controlled trial to compare an experimental intervention (early specific CBT including stress management and problem solving strategies with elements of mindfulness-based therapy (MBT) in a group setting) against unstructured group meetings. We hypothesised that subjects randomly allocated to the experimental intervention show less psychosocial impairment and specified affective symptomatology at 14 weeks compared to subjects receiving the control intervention.

## Methods/Design

### Design of the study

**Figure 1:**
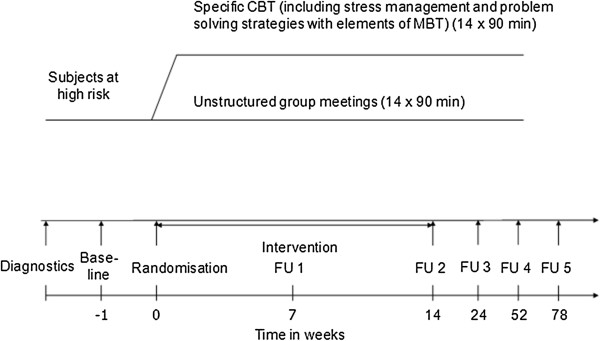
**Trial flow.** Legend: CBT (cognitive-behavioural psychotherapy), FU (follow-up).

This is a randomised controlled multi-centre clinical trial to evaluate the efficacy and safety of a specific CBT for young people at-risk for BD versus unstructured group meetings for 14 weeks each with long-term follow-up until week 78. Figure 1 summarises the trial flow. Study subjects, outcome assessors, and the statistician are blinded to treatment allocation. The study is conducted according to good clinical practice (GCP) standards and has been approved by the responsible local ethical committees.

### Description of intervention and control condition

In the absence of a published manual, an intervention was specifically designed to meet the needs of the bipolar high-risk clientele (*BEsT (be)for(e) Bipolar*, © C Marx, K Leopold and A Pfennig 2010). It consists of specific CBT, including stress management and problem solving strategies with elements of MBT in a group setting. The newly developed intervention was based on the manual ‘Cognitive psychoeducational therapy for bipolar disorders’ by Schaub *et al*. [28]; modules for stress management and problem solving strategies from the manual ‘Kognitiv-verhaltenstherapeutisches Behandlungsmanual’ by Meyer and Hautzinger [29] were integrated. Additionally, mindfulness exercises from MBT [30] are used. The aforementioned manuals were chosen by means of aptitude for the purpose of the intervention and availability of validation data. Modules were adapted to the needs of at-risk subjects similarly to the approach described by Bechdolf and Juckel [31] as some modules conceptualised for patients with full manifestation were weakened and terms of probability/risk were emphasised. Treatment modules of *BEsT (be)for(e) Bipolar*© include psycho-education about mental illnesses, and BD in particular, including treatment options, handling of early warning signs and crisis planning for prophylaxis, structuring of activities, cognitive strategies, and sensitisation for a balanced life rhythm. The control condition consists of unstructured group meetings where therapists are instructed to avoid therapeutic measures in any way possible. Both interventions are applied in groups of four to five subjects and in the same frequency and duration with 14 sessions each of 90 minutes within 14 weeks (one per week). To increase transparency and documentation as well as for analysis and comparability, sessions are videotaped.

### Setting and participants

Study participants were initially recruited at five, now at seven participating university centres which provide in- and outpatient care for patients with unipolar depressive and bipolar disorders (Department of Psychiatry and Psychotherapy, University Hospital Carl Gustav Carus Dresden; Department of Psychiatry, Ruhr University Hospital Bochum; Department of Psychiatry and Psychotherapy, University Medical Center Hamburg-Eppendorf; Department of Child and Adolescent Psychiatry and Psychotherapy, LWL University Hospital Hamm; Department of Psychiatry, University Hospital Cologne; Department of Psychiatry and Psychotherapy, University Hospital Würzburg, Department of Psychiatry, Psychosomatics and Psychotherapy, Charité-University Medicine, CCM; all Germany). Most of them additionally run early recognition centres for psychoses and/or affective disorders.

Key inclusion criteria for subjects include:

• Positive family history for affective and/or schizoaffective disorders (first and/or second degree relative)

• Reduction in psychosocial functioning/coping with demands of daily living (measured by the Social Interview Schedule, SIS, [32], German version: [33]) in the last 12 months compared to the previous 12 months

• Specified affective symptomatology (sub-threshold mania and/or at least sub-threshold depression with cyclothymic features and/or cyclothymic features, definitions see below) (measured by the Early Phase Inventory for bipolar disorders (EPI*bipolar*, ©Pfennig and Leopold 2010, [19]) and the Bipolar Prodrome Symptom Scale (BPSS)-Prospective (©C Correll 2007, [20]) in the last 12 months compared to the previous 12 months

• Aged 15 to 30 years

• Language capacity to take part in the trial

• Written informed consent to participate in the study.

Definitions of specified affective symptomatology are similar to those proposed by Bechdolf *et al*. [17]: sub-threshold mania is defined as a period of at least two consecutive days of abnormally and persistently elevated, expansive or irritable mood plus at least two of the following criteria: inflated self-esteem or grandiosity, decreased need for sleep, more talkative than usual or pressure to keep talking, flight of ideas or subjective experience that thoughts are racing, distractibility, increased goal-directed activity or psychomotor agitation. At least sub-threshold depression is defined as depressed mood or loss of interest or pleasure plus at least two of the following criteria: fatigue or loss of energy, feelings of worthlessness or excessive or inappropriate guilt, insomnia or hypersomnia nearly every day, psychomotor retardation or agitation, diminished ability to think or concentrate, recurrent thoughts of death/recurrent suicidal ideation or significant weight loss over a period of at least one week. Cyclothymic features are defined as numerous episodes with sub-threshold manic symptoms not meeting the definition of sub-threshold mania and numerous episodes with depressive symptoms.

Key exclusion criteria include:

• A history of treated or untreated manic episode of at least four days duration (Structured Clinical Interview for *Diagnostic and Statistical Manual of Mental Disorders, 4th edition* (DSM-IV) Disorders, SCID, in German: [34])

• A history of treated or untreated psychosis of at least seven days duration (SCID)

• Main symptomatology must not be present solely within the context of personality disorder or cyclothymia (SCID)

• Organic brain disorder

• Acute suicidality

• Severe, unstable medical condition (for example, cancer, neurological diseases)

• Intake of psychotropic medication (only medication for sleep disturbances and a stable antidepressant medication with serotonin reuptake inhibitors (SSRIs), venlafaxine, duloxetine, mirtazapine or valdoxan (stable means intake for at least eight weeks) are allowed, all other drug doses must be tapered-down and stopped before randomisation).

Rationale for choosing the presented inclusion and exclusion criteria are as follows. First degree relatives of affected individuals have a ten-fold increased risk to also develop the disease compared to relatives of unaffected controls [35]. Twin and adoption studies have provided compelling evidence for heritable factors playing a major role in the pathogenesis of BD (see [36]). A genetically enriched population with first affective symptoms that already impact psychosocial functioning seemed to us the best way to identify subjects at high risk for developing BD. Participants must not already be diagnosed as having unipolar or bipolar affective disorders but can be diagnosed as having cyclothymia, attention deficit/hyperactivity disorder (ADHD), personality disorder or psychosis of less than seven days duration, if the symptomatology is not explained solely by this diagnosis. In view of the often unspecific presentation of high-risk subjects, this ensures reduction of false-negative recruitment, while at the same time of course the risk for false-positive enrolment is increased. The pilot findings by Bechdolf *et al*. [17,18] which indicated conversion rates to four days of mania in up to 30% within 12 months, depending on the criteria applied, support this approach of inclusion criteria definition. The diagnostic procedure comprises using the SCID plus the result of a consensus board of two clinical psychiatrists per centre.

Subjects in both treatment groups are allowed to use unstructured consultations with their usual treating physician at any time. After the intervention period, subjects in both groups are allowed to use unstructured psychotherapy sessions and psychopharmacotherapy if needed (restricted to intermittent symptomatic treatment of unspecific symptoms such as sleep disturbances and anxiety/agitation administered no longer than seven days). As mentioned, a stable antidepressant medication with SSRI, venlafaxine, duloxetine, mirtazapine or valdoxan (stable means intake for at least eight weeks) is allowed at randomisation and can be prolonged during the study. Formal, structured psychotherapy and psychopharmacotherapy other than described before results in study drop-out.

As mentioned in the inclusion criteria, written informed consent is obtained from each subject after thorough information about the study has been provided.

### Outcome measures

Primary efficacy endpoints:

• Psychosocial functioning/coping with demands of daily living (SIS) at 14 weeks

• Specified affective symptomatology (Hamilton Rating Scale for Depression (HAMD, [37]), Young Mania Rating Scale (YMRS, [38]), EPI*bipolar*, BPSS) at 14 weeks

Key secondary endpoints:

• SIS at 7, 24, 52 and 78 weeks

• Mini version of the International Classification of Functioning, Disability and Health for Mental Disorders (MINI-ICF-P, [39,40]) at 7, 14, 24, 52 and 78 weeks

• Specified affective symptomatology (HAMD, YMRS, EPI*bipolar*, BPSS) at 7, 24, 52 and 78 weeks

• Perception of, reaction to and coping with stress: Alltags-Belastungs-Fragebogen (ABF, [41]), Trierer Inventar zum chronischen Stress (TICS, [42]), Stress-Reaktivitäts-Skala (SRS, [43]), Fragebogen zum Umgang mit Belastungen im Verlauf (UBV, [44]), Fragebogen zur Erfassung von Ressourcen und Selbstmanagementfähigkeiten (FERUS, [45]) at 7, 14, 24, 52 and 78 weeks

• Development of bipolar disorders at 52 and 78 weeks (SCID).

Assessment of safety: structured assessment of adverse events (GCP standard, interview: description of type, severity and relation to intervention). See Table 1 for an overview of instruments applied.

**Table 1:** Instruments applied at individual study visits

** Time**	**Baseline**	**FU 1**	**FU 2**	**FU 3**	**FU 4**	**FU 5**
**Instruments**	**Wk -1**	**Wk 7**	**Wk 14**	**Wk 24**	**Wk 52**	**Wk 78**
SIS	X	X	X	X	X	X
MINI-ICF-P	X	X	X	X	X	X
HAMD	X	X	X	X	X	X
YMRS	X	X	X	X	X	X
EPI*bipolar*	X	X	X	X	X	X
BPSS	X	X	X	X	X	X
ABF	X	X	X	X	X	X
TICS	X	X	X	X	X	X
SRS	X	X	X	X	X	X
UBV	X	X	X	X	X	X
FERUS	X	X	X	X	X	X
SCID	X				X	X

Legend: FU, follow-up; Wk, week; SIS, Social Interview Schedule, English version: [32], German version: [33]); MINI-ICF-P, Mini version of the International Classification of Functioning, Disability and Health for Mental Disorders, [39,40]; HAMD, Hamilton Depression Symptom Scale 17 item version, [37]; YMRS, Young Mania Rating Scale, [38]; EPI*bipolar*, Early Phase Inventory for bipolar disorders, ©Pfennig and Leopold 2010, [19]; BPSS, Bipolar Prodrome Symptom Scale-Prospective, ©C Correll 2007, [20]; ABF, Alltags-Belastungs-Fragebogen, [41]; TICS, Trierer Inventar zum chronischen Stress, [42]; SRS, Stress-Reaktivitäts-Skala, [43]; UBV, Fragebogen zum Umgang mit Belastungen im Verlauf, [44]; FERUS, Fragebogen zur Erfassung von Ressourcen und Selbstmanagementfähigkeiten, [45]; SCID, Structured Clinical Interview for DSM-IV Disorders, in German: [34]. The baseline time is week minus 1.

Rationale for choosing the presented outcome measures are as follows. We chose coping with demands of daily living as one primary outcome measure as it is a sensitive measure of changes in psychosocial functioning that in turn highly influences the long-term outcome of the disorder. Our study population consists of subjects at high risk for developing BD; they do not fulfill the diagnosis (yet) but already show impairment of psychosocial functioning. Improving this should have a great impact on the course of the disease development either by postponing/alleviating or even preventing the full-blown disorder. Additionally, we use the change in affective symptomatology as a primary endpoint since psychosocial functioning is only in part explained by psychiatric symptomatology.

To get an idea about adherence on the therapist’s part to the treatment manual or instructions regarding the control group setting, videotaped treatment sessions which have been developed based on the German adapted version of the Cognitive Therapy Scale for Psychosis [46] by Wittorf *et al*. [47], will be reviewed. Adherence on the subject’s part is assessed by checking on participation in the sessions as well as home work completion in the active intervention groups.

### Blinding and methods against bias

An independent statistician uses a centrally computer-generated block-designed randomisation procedure stratified by centre. Only the principal investigator of the study (AP) and the psychotherapist of the individual centre are notified of the randomisation result; subjects and outcome assessors are kept blinded. The statistician who will later analyse the study results is kept blinded throughout the whole study.

An intensive three days training of GCP, study procedures and applied instruments was provided to psychotherapists and raters before onset of the study to minimise differences between centres. Regular trainings are provided throughout the study. All group sessions (in the intervention and control groups) are videotaped and stored for analysis. The trial is managed with help of the departmental clinical trial centre.

To ensure the performance according to the study protocol and the quality of the study monitoring is performed in each study centre at appointed time intervals and as needed (at least per centre once at initiation of the study, once after the first subject was randomised, once after 1/3, 2/3 and 3/3 of subjects were recruited, and once when the last subject completed the study), the monitoring process is performed according to prepared standard operating procedures (SOPs). The monitor checks completeness and plausibility of the data and aligns the study data to the original data (Source Data Verification). This is accomplished by accessing the original subjects’ charts; the subjects will give their permission to that procedure within the informed consent form. The monitor ensures the basic claim of integrity and protection of the subjects’ privacy. If not all data are examined, the monitor must justify that procedure and must pull a proper random sample for the check.

Supervision of the trial is present in the form of a scientific advisory board including members especially experienced in assuring data quality and safety. They also supervised the development of the application protocol and the progress of the trial including decision making on whether to perform interim analyses or modify/stop the trial in cases of unforeseen problems arising.

### Power

Sample size for the ANCOVA will be approximated with the two-sided unpaired *t*-test. The primary outcome measure is the difference in the adapted overall social maladjustment score. Here, the number of items in the categories M and S with a rating of 2, 3 or 4 is divided by the number of applicable items in the categories M and S. With an estimated effect size of about 0.5, a sample size of 2 times 50 patients is required to show this difference with a power of 80% and an alpha of 5% (calculated with G*Power 3.1.2).

We assume that the compliance will be relatively good here since the intervention does not result in major side effects and the subjects already experience symptoms. However, we assume conservatively that there will be about a 30% drop-out rate, an estimate that was accounted for in the sample size estimation. Follow-up of subjects that drop out of the study will last for the planned duration of the study.

### Statistical analysis

Efficacy: two-group comparison of the psychosocial functioning/coping with demands of daily living (SIS, MINI-ICF-P), specified affective symptomatology including mood swings (HAMD, YMRS, EPI*bipolar*, BPSS-P) and difference in perception of, reaction to and coping with stress (ABF, TICS, SRS, UBV, FERUS) at 7, 14, 24, 52 and 78 weeks with baseline values as covariates. In all cases, the 14-week value is the primary time-point of interest. A repeated measures analysis will be carried out as a secondary result.

Description of the primary efficacy analysis: ANCOVA with SIS at 14 weeks and separately with specified affective symptomatology at 14 weeks as dependent variable and baseline SIS, affective symptomatology, centre, age, sex, education and group as covariates. We will also look into influence of the therapist on results by additionally including that variable, if not highly correlated with the variable centre, into the ANCOVA. Population: intention-to-treat (ITT) analysis with last observation carried forward (LOCF) in case of missing values.

Secondary endpoints:

• Difference between the groups in MINI-ICF-P at 7, 14, 24, 52 and 78 weeks with baseline values as covariates

• Change in specified affective symptomatology including mood swings at 7, 24, 52 and 78 weeks compared to baseline within each group

• Difference between the groups in perception of, reaction to and coping with stress at 7, 14, 24, 52 and 78 weeks with baseline values as covariates

• Difference between the groups in SIS at 7, 24, 52 and 78 weeks with baseline values as covariates

• Difference between the groups in rates of developing bipolar disorders at 52 and 78 weeks

Safety: assessment of frequency and type of unwanted effects at 7, 14, 24, 52 and 78 weeks compared to baseline within the groups and difference between the groups.

The study was approved by the respective ethics committees of all participating study centres (see Table 2).

**Table 2:** Ethics committees of the participating study centres

**Ethics committee**	**Reference number**
Medizinische Fakultät, TU Dresden	EK 60022010
Medizinische Fakultät, Ruhr University Bochum	3757-10
Ärztekammer Hamburg	MC-196/10
Medizinische Fakultät, Ruhr University Bochum	3782-10
Medizinische Fakultät der Universität zu Köln	10-164
Medizinische Fakultät, University Würzburg	204/12_z
Ethikkommission der Charité Berlin	EA1/233/12

## Discussion

To our knowledge, this is the first study to evaluate early specific cognitive-behavioural psychotherapy in subjects at high risk for BD. We hypothesise that subjects randomly allocated to early specific CBT, including stress management and problem solving strategies with elements of MBT in a group setting, show less reduction in psychosocial functioning and less specified affective symptoms at 14 weeks compared to subjects receiving unstructured group meetings.

### Limitations

One possible limitation of external validity relates to recruitment in university hospitals in urban areas of Germany and selection of patients that utilise the healthcare system or an early recognition centre. People who do not seek (medical) advice and help may show different characteristics and worse treatment effects.

Diagnostic measures in early stages of disease development may show limited precision. The predictive validity of the used at-risk state definitions are currently assessed in validation studies (for example, [20]). Consequences of false positive ‘diagnoses’ may result in additional psychosocial impairment, including worry about possible psychiatric disease and unneeded treatment causing preventable adverse drug reactions and/or adverse effects of interventions. However, CBT as a non-pharmacological intervention with a low risk of adverse events seems to be appropriate from the risk-benefit perspective.

The follow-up time of the study is restricted to 78 weeks per individual, which will not be long enough to detect all cases of conversion to manifest disorder. Each participating centre agreed to extend the follow-up time to as long as possible within their clinical or early recognition centre routine.

The therapeutic manual was only developed shortly before starting the study, so its efficacy had not been previously assessed. Feasibility was tested in the early recognition centre in Dresden; however, use in a multi-centre study is only practiced within the present study.

Naturally, the therapist can not be blinded to type of intervention. The subjects are kept blinded and are only sketchily informed about the content and design of the intervention to be studied.

We decided against a third study arm providing a wait-list control condition. Therefore we cannot measure the effect of meeting in a group *per se*.

To reach the estimated sample size, recruitment processes were established and interconnections between departments and early detection services with local services and each other were strengthened. Continuous outreach work is performed.

### Strengths

The study has several major strengths: the high-risk status is assessed and followed-up by the currently relevant diagnostic instruments available to the investigators. Also, the study is timely: treatment options for the high-risk clientele are needed with subjects suffering from impairment. Here, early on, one strategy is scientifically assessed for efficacy and safety. In a randomised controlled fashion with blinded subjects, outcome assessors and statistician, the control condition is matched as far as possible to the intervention with regard to frequency and duration, group character and participation of a trained psychologist or psychotherapist. Trainings and monitoring for therapists and raters are conducted on a regular basis.

The multi-centre character of the study increases sample size and generalizability of the results. The cooperating centres in the study are leading clinical and research centres for high-risk individuals for psychosis and BD. In 2009, they founded the ‘Network for Early Recognition and Intervention in Bipolar Disorders’ (NERIBID, [48]). The aim of the network is to cooperatively develop and perform research projects and to develop clinical and research standards for our centres.

### Relevance of study for clinical practice and future research

Our approach to identify patients with a positive family history in potentially early stages of BD is comparable to that in high-risk subjects for psychosis. In the present study, clinical presentation, impairment of psychosocial functioning, and family history is combined to define persons at risk of a significant conversion risk.

Studies show that cognitive-behavioural psychotherapy (CBT) is effective in patients with BD in reducing symptomatology. Scott *et al*. [27] showed that although there was no significant difference in overall recurrence rates, CBT was more effective than treatment as usual in patients with few compared to those with many recurrent bipolar episodes in their history. We therefore suggest that the intervention might be more effective in the early stages of disease and so even more in the prodromal phase. In persons at risk for psychosis, CBT was shown to be effective in alleviating symptomatology. The transition risk was reduced as long as the intervention (psychopharmacological and psychotherapeutic) could reduce the symptomatology. After the end of the intervention however, general transition rates increased and then almost equalled that of the group without intervention; see [49-53]. There were however, as stated above, enduring effects of CBT in that the likelihood of being prescribed antipsychotics in the three-year follow-up was reduced ([50]). Interestingly, in the group of subjects with psychological vulnerabilities targeted by the intervention, the transition risk to psychosis was also significantly reduced [49,50]. In full-blown schizophrenia, of course, CBT without psychopharmacology would be not effective enough [54].

Additionally, we suggest that the components of the studied intervention tackle symptomatology associated with the factors that influence components of the vulnerability-stress-system (for example, sleep regulation, stress management and handling of personality features) and, therefore, should be best suited to alleviate/postpone or even prevent onset/conversion to BD. Most importantly, evidence of the effectiveness and adverse events of CBT in subjects at high risk for BD is missing so that our study will answer this clinically relevant question regardless of the study results.

If efficacy of early specific CBT can be demonstrated by our trial, the level of evidence of the treatment of subjects at high risk for BD will be significantly raised. Subsequently, future research should adopt and evaluate the effectiveness of such a tailored intervention in less highly-selected populations in routine care. The results of the proposed study could be used to establish preventive strategies for BD that are adequate from the risk-benefit perspective.

## Trial status

Ongoing, start of recruitment: August 2010, first patient: October 2010, 50% randomised: March 2013.

## Abbreviations

ABF: Alltags-Belastungs-Fragebogen; ADHD: attention deficit/hyperactivity disorder; BD: bipolar disorder; BPSS: Bipolar Prodrome Symptom Scale-Prospective; CBT: cognitive-behavioural psychotherapy; DSM-IV: *Diagnostic and Statistical Manual of Mental Disorders, 4th edition*; EPIbipolar: Early Phase Inventory for bipolar disorders; FERUS: Fragebogen zur Erfassung von Ressourcen und Selbstmanagementfähigkeiten; FU: follow-up; GCP: good clinical practice; HAMD: Hamilton Depression Symptom Scale 17 item version; ITT: intention-to-treat analysis; LOCF: last observation carried forward; MBT: mindfulness-based therapy; MINI-ICF-P: Mini version of the International Classification of Functioning, Disability and Health for Mental Disorders; NERIBID: Network for Early Recognition and Intervention in Bipolar Disorders; SCID: Structured Clinical Interview for DSM-IV Disorders.

## Competing interests

The authors declare that they have no competing interests.

## Authors’ contributions

AP: conception and design of the study, data collection, manuscript writing and final approval of the manuscript. KL: conception and design of the study, data collection, critical revision and final approval of the manuscript. AB: design of the study, data collection, critical revision and final approval of the manuscript. CC: design of the study, critical revision and final approval of the manuscript. MH: data collection, critical revision and final approval of the manuscript. GJ: conception and design of the study, data collection, critical revision and final approval of the manuscript. ML: data collection, critical revision and final approval of the manuscript. CM: data collection, critical revision and final approval of the manuscript. TDM: design of the study, critical revision and final approval of the manuscript. SP: data collection, critical revision and final approval of the manuscript. AR: data collection, critical revision and final approval of the manuscript. MRW: data collection, critical revision and final approval of the manuscript. NMS: data collection, drafting and final approval of the manuscript. TS: data collection, critical revision and final approval of the manuscript. MB: conception and design of the study, critical revision and final approval of the manuscript. All authors read and approved the final manuscript.

## Authors’ information

Andrea Pfennig and Karolina Leopold share the first authorship.
